# Synergistic neoadjuvant radioimmunotherapy in locally advanced rectal cancer: mechanisms of pathologic response and the shift toward organ preservation

**DOI:** 10.3389/fimmu.2026.1832333

**Published:** 2026-07-15

**Authors:** Shu Yan, Guangchao Zhang, Xiaojing Jia

**Affiliations:** 1 Department of Tumor Radiotherapy, Jilin City Hospital of Chemical Industry, Jilin, China; 2 Department of Tumor Radiotherapy, The Second Hospital of Jilin University, Changchun, China

**Keywords:** immune checkpoint inhibitors, locally advanced rectal cancer, organ preservation, pathological complete response, radioimmunotherapy

## Abstract

The management of locally advanced rectal cancer (LARC) has progressively shifted beyond surgery alone toward integrated protocols that include neoadjuvant chemoradiotherapy (nCRT). Even so, the risks of distant metastasis and organ dysfunction remain significant challenges. It is against this backdrop that immune checkpoint inhibitors (ICIs) combined with chemoradiotherapy have begun to redefine therapeutic possibilities. Radiotherapy (RT) induces immunogenic cell death (ICD), enhances antigen presentation, and activates systemic immune responses, whereas ICIs overcome immune suppression by blocking pathways such as PD-1/PD-L1. This synergistic approach converts immunologically cold tumors into infiltrated, hot phenotypes. Building on this rationale, recent Phase II trials have explored immune consolidation following short-course radiotherapy (SCRT) within a total neoadjuvant therapy (TNT) framework. What emerged was particularly compelling: pathological complete response (pCR) rates rose notably, in some cases doubling historical benchmarks. This improvement is not merely a numerical gain; it translates into tangible clinical opportunities, including organ preservation and the feasibility of watch-and-wait (W&W) protocols for selected responders. Notably, SCRT appears especially compatible with subsequent immunotherapy, perhaps due to its abbreviated yet potent immunomodulatory effects, offering a pragmatic alternative to conventional long-course regimens. Looking ahead, the push toward personalization is gaining momentum. Biomarkers like MMR/MSI status, features of the tumor immune microenvironment, and dynamic changes in ctDNA are increasingly guiding trial design and treatment sequencing. While promising, these tools require further validation in larger, more diverse cohorts. Ultimately, the central question remains: how can we convert higher pCR rates into lasting survival benefits while safeguarding quality of life? Answering this will depend on rigorously designed Phase III trials, thoughtful integration of predictive biomarkers, and continued refinement of how and when we combine radiation, chemotherapy, and immunotherapy. Only then can we ensure that advances in biology translate into meaningful human outcomes.

## 1 Introduction

LARC is defined as malignant rectal tumors that have penetrated the muscularis propria of the intestinal wall (T3/T4 stage) or involve regional lymph node metastasis (N+), but without distant metastasis (M0). It accounts for approximately 35%–40% of newly diagnosed rectal cancer cases (1). Due to its therapeutic complexity and challenges, this disease represents a significant clinical challenge in gastrointestinal oncology. While the traditional standard treatment regimen of nCRT followed by total mesorectal excision (TME) has markedly improved local control, reducing the 5-year local recurrence rate from historically >25% to approximately 5%–10% (2), it has failed to effectively curb systemic dissemination. Up to 25%–30% of patients ultimately develop distant metastases, which remains the primary threat to long-term survival (3, 4). Furthermore, surgery for low rectal cancer often results in permanent stoma formation, severely impacting quality of life (5, 6).

Against this backdrop, the core objective for improving the prognosis of LARC patients lies in further enhancing treatment response rates, reducing the risk of distant metastasis, and preserving organ function to the greatest extent possible. pCR, defined as the complete disappearance of the tumor following neoadjuvant therapy, has been demonstrated to correlate closely with lower local recurrence rates, superior long-term survival, and a higher likelihood of sphincter preservation (7). Therefore, improving pCR rates not only enhances oncological outcomes but also provides a critical opportunity for non-surgical treatment and organ preservation in some patients. To systematically address the risk of distant metastasis and maximize tumor regression, thereby increasing pCR rates and organ preservation opportunities, the TNT strategy has emerged. By completing all chemotherapy and RT preoperatively, TNT aims to eradicate micrometastases, maximize tumor regression, and establish a foundation for implementing a W&W strategy in eligible patients (8). However, even with TNT, pCR rates remain between 20% and 40% (9, 10). This challenge stems largely from the fact that over 90% of LARC tumors exhibit proficient mismatch repair (pMMR)/microsatellite stable (MSS) characteristics. Their immunosuppressive cold tumor microenvironment limits response to conventional single-agent immunotherapy, thereby constraining further improvement in treatment efficacy (11).

Faced with the dual challenges of distant metastasis risk and organ dysfunction, coupled with the efficacy bottleneck of TNT strategies in pMMR/MSS LARC, researchers have turned their attention to the highly promising approach of combining ICIs within the TNT framework. Encouragingly, multiple breakthrough studies in recent years have consistently demonstrated that ICIs combined with TNT regimens significantly enhance pCR rates (12–14), offering unprecedented hope for overcoming current treatment limitations and achieving higher rates of organ function preservation. Against this backdrop, this review aims to systematically analyze the mechanistic basis of immune combination strategies (ICIs + chemoradiotherapy) in LARC treatment; comprehensively review breakthrough clinical evidence demonstrating their potential to induce high pCR rates and organ preservation; thoroughly explore key strategies for related safety management; and prospectively outline future optimization directions and challenges. By integrating current evidence and insights, we seek to inform clinical decision-making and further progress toward the dual goals of survival benefit and organ preservation for patients with LARC.

## 2 Mechanistic basis of immunotherapy combination therapy

**Figure 1:**
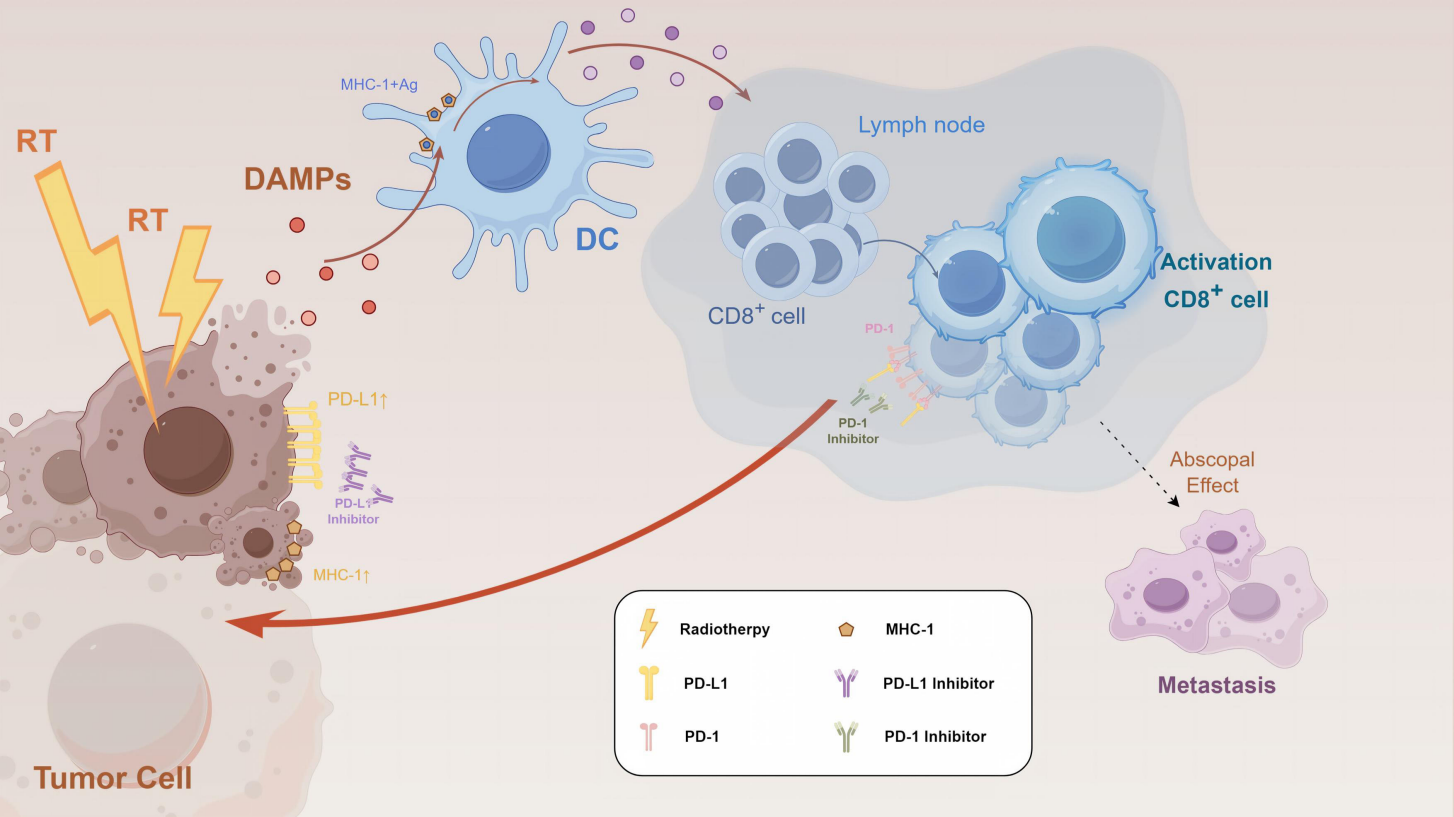
Schematic of the radiotherapy-immunotherapy synergy mechanism. RT induces ICD, releasing DAMPs and neoantigens that activate DCs and prime CD8^+^ T cells. RT upregulates MHC−I and PD−L1 on tumor cells (↑). Activated DCs present antigens via MHC−I to CD8^+^ T cells, leading to T-cell activation. The PD−L1/PD−1 pathway suppresses T cells, but ICIs block this inhibition. Effector T cells kill tumor cells locally. A rare abscopal effect (regression of distant tumors) has been reported occasionally after RT alone but is exceptional; ICIs may increase its likelihood.Abbreviations: DAMPs, damage−associated molecular patterns; DC, dendritic cell; ICD, immunogenic cell death; ICI, immune checkpoint inhibitor; MHC−I, major histocompatibility complex class I; PD−1, programmed cell death protein 1; PD−L1, programmed death−ligand 1; RT, radiotherapy.

The introduction of immunotherapy into LARC neoadjuvant treatment is grounded in the core theoretical principle that RT and ICIs exhibit significant synergistic effects (**Figure 1**). This synergy is not merely an additive effect but stems from complementary biological mechanisms in activating and enhancing antitumor immune responses (15).

Traditionally, RT has been regarded as a localized treatment that kills tumor cells by directly damaging their DNA. However, recent research has revealed its pivotal role in activating systemic antitumor immunity, centered on inducing an *in situ* vaccine effect. This effect originates from radiation-induced ICD, during which massive tumor antigens and endogenous danger molecules are released as potent danger signals that strongly attract and activate antigen-presenting cells, particularly dendritic cells (DC) (16).

At the molecular level, radiation-induced DNA damage not only increases mutational load but also generates cytosolic double-stranded DNA (dsDNA) fragments through misrepair or replication stress. These dsDNA fragments are recognized by the cyclic GMP-AMP synthase (cGAS), which catalyzes the production of 2’3’-cyclic GMP-AMP (cGAMP), thereby activating the stimulator of interferon genes (STING) pathway (17). STING signaling triggers a type I interferon (IFN-I) response that is essential for DC activation, cross-priming of CD8^+^ T cells, and the generation of systemic antitumor immunity (17, 18). Recent evidence has directly implicated the cGAS-STING/type I IFN axis as a key determinant of response to SCRT in rectal cancer (19). However, the exonuclease three-prime repair exonuclease 1 (TREX1) degrades cytosolic dsDNA, thereby limiting cGAS-STING activation (20). TREX1 is induced by radiation doses above 12–18 Gy in various cancer cell lines, and its expression attenuates the immunogenicity of irradiated tumors (18, 20, 21). This dose-dependent effect has major clinical implications: high-dose per fraction (e.g., ablative doses >12–18 Gy) may suppress the abscopal effect by TREX1-mediated DNA degradation, whereas doses below this threshold (e.g.,≤10 Gy per fraction) are more likely to preserve the cGAS-STING pathway and promote adaptive immunity (18, 20, 21). Importantly, while both SCRT (5 × 5 Gy, 5 Gy per fraction) and long-course RT (LCRT, 1.8–2 Gy per fraction) remain below the TREX1 threshold, higher doses per fraction have been shown to induce increased levels of ICD markers, antigen release, and damage-associated molecular patterns (DAMPs) in a dose-dependent manner (22). These molecular considerations indicate that SCRT possesses a distinct dose-fractionation profile, as it is high enough per fraction to trigger potent ICD and antigen release while remaining below the TREX1 threshold to avoid immunosuppression. This profile distinguishes SCRT from conventional LCRT and provides a biological rationale for comparing their immunomodulatory effects in combination with ICIs.

These events collectively establish an *in situ* vaccine environment within the tumor site, transforming the tumor itself into a highly immunogenic antigen reservoir and laying the foundation for systemic immune responses (23, 24). Building upon this, RT further drives the expansion of local immune responses into systemic activity through antigen cross-presentation by DC and T-cell activation. Released tumor antigens and DAMPs are captured and processed by DC within the tumor microenvironment, prompting their maturation and migration to draining lymph nodes (24). Within lymph nodes, these cells present antigens to CD8^+^ and CD4^+^ T cells via major histocompatibility complex class I (MHC-I) and class II (MHC-II) molecules, respectively, thereby initiating antigen-specific T-cell immunity. Additionally, RT significantly upregulates the expression of multiple immune-related molecules on the surfaces of tumor cells and antigen-presenting cells (25, 26). Among these, enhanced expression of MHC-I molecules is crucial for effectively activating CD8^+^ T cells (27). Meanwhile, interferon pathway-mediated upregulation of programmed death-ligand 1 (PD-L1), while serving as a tumor immune escape mechanism, also provides a potential target for combination therapy with ICIs (28, 29). Ultimately, these mechanisms are thought to contribute to the so-called abscopal effect, a rare phenomenon in which tumors regress in distant, non-irradiated sites following local irradiation (30, 31). However, it should be noted that the abscopal effect remains a medical enigma in clinical practice and has been observed only in isolated case reports and occasional series, particularly in the context of RT alone. Recent research evidence suggests that combining RT with ICIs may increase the likelihood of such systemic responses, although the abscopal effect remains far from a predictable or universal outcome (32). The multidimensional remodeling of the tumor microenvironment by RT, including the promotion of T−cell chemokines, the modulation of tumor−associated macrophages, and the improvement of tumor vascular permeability, may theoretically support the activation and migration of systemic T cells. However, there is currently insufficient clinical evidence to demonstrate a consistent synergistic effect in unselected patients receiving RT, with or without immunotherapy. Therefore, although the immunomodulatory potential of RT from local initiation to systemic control, is biologically plausible, its translation into clinically meaningful abscopal responses remains extremely rare and warrants further investigation.

However, RT-induced immune activation is a double-edged sword. While releasing antigens and activating T cells, RT can also reshape an adaptive immunosuppressive microenvironment by upregulating immune checkpoint molecules (such as PD-L1) on tumor cells or antigen-presenting cells, recruiting regulatory T cells (Tregs), and promoting the release of immunosuppressive cytokines. This limits the intensity and persistence of the antitumor immune response. This represents the critical target for ICIs to exert synergistic effects. The core immunological mechanism of ICIs lies in blocking inhibitory signaling pathways like PD-1/PD-L1, thereby releasing T-cell functional suppression and unleashing effector T cells and tumor-infiltrating lymphocytes activated by RT (33). This blockade not only significantly enhances local tumor cell clearance but also promotes clonal expansion of effector T cells and the formation of memory T cells, laying the foundation for sustained systemic immune surveillance (34). Thus, the incorporation of ICIs not only amplifies the acute immune effects of RT but may also achieve sustained control over micrometastases by shaping long-term immune memory. This theoretically provides an immunological basis for reducing the distant metastasis rate in LARC.

Crucially, this combined strategy offers an immunological solution for overcoming pMMR/MSS cold tumors, which constitute the vast majority of LARC cases. RT effectively transforms immune desert or immune-rejecting tumor microenvironments into immune-infiltrated states through the aforementioned mechanisms (ICD, antigen release, MHC-I upregulation, and chemokine induction), thereby increasing the infiltration and function of CD8^+^ T cells within tumors (35, 36). ICIs, by lifting immune suppression, stabilize and amplify this heating effect, enabling pMMR/MSS tumors, originally resistant to immunotherapy, to benefit from combination therapy, thereby achieving a breakthrough increase in pCR rates (37).

## 3 Exploration and preliminary efficacy of immunotherapy combination strategies

This review systematically retrieved multiple published pivotal Phase II/III studies from databases including PubMed, Embase, and the Cochrane Library, as well as abstracts from conferences such as ASCO and ESMO. Despite heterogeneity in the timing of ICI integration, RT regimens, and combinations with chemotherapy/immunotherapy agents, existing evidence consistently demonstrates that adding ICIs to nCRT significantly enhances pathological response rates in LARC, with overall manageable safety profiles. Key study data are summarized in the table below (**Table 1**).

**Table 1:** Summary of key clinical trials on immune-combination neoadjuvant therapy for locally advanced rectal cancer.

Trial(Year)	ID	Phase	Regimen	n	pCR (%)	MPR (%)	cCR (%)	≥G3 AEs	Key population
RAPIDO (2020)	NCT01558921	III	EA: SCRT → CAPOX/FOLFOX4 → TME/W&W CA: (LCRT + Capecitabine) → TME ± Adjuvant Chemo	462 450	28.0 14.0	/	/	48.0% 25.0%	Unselected
AVANA (2021)	NCT03299660	II	LCRT+ Capecitabine + Avelumab → TME	101	23.0	61.5	/	Non-irAEs: 8.0% irAEs: 4.0%	Primarily MSS
DUREC (2023)	EudraCT 2018-004835-56	II	mFOLFOX6 + Durvalumab → LCRT + Capecitabine + Durvalumab →TME	61	36.0	71.0	/	13.0%	MSS
NRG-GI002 (2023)	NCT02921256	II	PA: FOLFOX → LCRT + Capecitabine + Pembrolizumab → TME CA: FOLFOX → LCRT + Capecitabine → TME	90 95	31.9 29.4	/	/	48.2% 37.3%	Unselected
VOLTAGE-A (2023)	NCT02948348	II	MSS: LCRT + Capecitabine → Nivolumab →TME MSI-H: LCRT + Capecitabine → Nivolumab →TME	37 5	30.0 60.0	38.0 /	/	/	MSS/MSI-H
TORCH (2024)	NCT04518280	II	A: SCRT → CAPOX + Toripalimab → TME/W&W B: CAPOX + Toripalimab → SCRT → CAPOX + Toripalimab → TME/W&W	62 59	50.0 50.0	67.5 70.6	43.5 35.6	45.2% 42.4%	MSS
POLARSTAR(2024)	NCT05245474	II	ConA: LCRT+ Capecitabine + Tislelizumab → TME SA: LCRT + Capecitabine → Tislelizumab → TME CA: LCRT + Capecitabine → TME	59 55 57	27.1 32.7 14.0	59.0 56.0 40.0	/	3.0% 5.0% 0.0%	Primarily MSS
UNION (2024)	NCT04928807	III	EA: SCRT → CAPOX + Camrelizumab → TME CA: LCRT + Capecitabine → CAPOX → TME	113 118	39.8 15.3	/	/	29.2% 27.2%	Primarily MSS
NCT04304209 (2024)	NCT04304209	II	CA: LCRT + CAPOX EA: LCRT + CAPOX + Sintilimab	67 67	28.6 36.8	/	26.9 44.8	32.8%(Myelosupp.) 25.4%(Myelosupp.)	Unselected
PRECAM (2024)	NCT05216653	II	SCRT → CAPEOX + Envafolimab → TME	32	62.5	75.0	/	6.3%	MSS
STELLAR II (2025)	NCT05484024	II/III	iTNT: SCRT → CAPOX/mFOLFOX + Sintilimab → TME/W&W TNT: SCRT → CAPOX/mFOLFOX →TME/W&W	110 108	43.1 20.7	65.3 46.3	45.5 25.0	34.5% 19.4%	MSS
SPRING-01 (2025)	ChiCTR2100052288	II	EA: SCRT → CAPOX + Sintilimab → TME CA: SCRT → CAPOX → TME	49 49	59.2 32.7	73.5 46.9	61.2 32.7	33.0% 35.0%	Unselected

pCR, pathological complete response; MPR, major pathological response; cCR, clinical complete response; AE, adverse event; ≥G3 AEs, grade 3 or higher adverse events; irAEs, immune-related adverse events; Non-irAEs, non-immune-related adverse events; Myelosupp., myelosuppression; SCRT, short-course radiotherapy; LCRT, long-course chemoradiotherapy; CAPOX, capecitabine plus oxaliplatin; FOLFOX, fluorouracil, leucovorin, and oxaliplatin; TME, total mesorectal excision; W&W, watch and wait; EA, experimental arm; CA, control arm; PA, pembrolizumab arm (specific to NRG-GI002); ConA, concurrent immunotherapy arm; SA, sequential/consolidation immunotherapy arm; iTNT, immune-enhanced total neoadjuvant therapy; MSS, microsatellite stable; MSI-H, microsatellite instability-high.

“/” indicates data not reported or not applicable. Percentages represent incidence rates. For comparative AE data (e.g., 32.8% *vs* 25.4%), the format denotes experimental arm % *vs* control arm %.

Immunotherapy combination strategies have elevated the pCR rate in LARC to unprecedented levels. Among the predominant pMMR/MSS patient population, combination regimens based on SCRT demonstrated particularly outstanding outcomes: pCR rates reached 62.5% in the PRECAM study (38), 59.2% in the SPRING-01 study (39), and 50% in the TORCH study (13, 40, 41). The Phase III UNION study further confirmed that SCRT combined with immunochemotherapy significantly outperformed conventional long-course chemoradiotherapy in achieving pCR. Even sequential immunotherapy consolidation following LCRT, as demonstrated in the POLARSTAR study (42), markedly increased pCR rates from 14.0% to 32.7% (12). Moreover, the rate of major pathological response (MPR) typically increases substantially concurrently, indicating this strategy induces profound tumor regression. While achieving significant efficacy, the combined regimen demonstrated overall good tolerability without unexpected safety signals. Detailed safety and toxicity data, including treatment-related adverse events (AEs) and immune-related adverse events (irAEs), are summarized in **Table 1** and discussed in the Safety and Toxicity section below. Existing clinical evidence consistently demonstrates that the combination of ICIs with neoadjuvant therapy significantly improves the pCR rate in LARC, thereby establishing a solid foundation for organ-preserving strategies. However, variations in efficacy across different studies suggest that treatment outcomes may be influenced by multiple factors, including the timing of immune integration, RT fractionation schemes, and patient selection. The following sections will delve into these three core optimization directions.

### 3.1 Optimizing the sequence of immunotherapy integration: synchronized, sequential, or induction?

The timing and sequence of integrating ICIs with conventional nCRT represent critical strategic variables influencing the intensity of antitumor immune responses and clinical outcomes. Current clinical exploration primarily follows three integration hypotheses and pathways: Concurrent integration aims to synchronously block immunosuppressive pathways during the RT-induced *in situ* vaccine window to synergistically activate T-cell responses (43, 44). Sequential or consolidation approaches advocate first reshaping the tumor microenvironment through chemoradiotherapy, followed by ICI administration to eliminate residual disease and establish immune memory (45, 56). Induction strategies attempt to first reverse systemic immunosuppression, thereby creating favorable conditions for subsequent localized treatment (47, 48).

**Figure 2:**
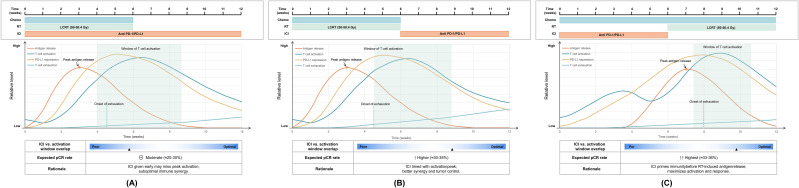
Timeline of tumor microenvironment dynamics during LCRT−based neoadjuvant regimens. The dynamic curves are qualitative, literature−informed representations derived from published biological observations and the known treatment timeline of LCRT. They are intended to illustrate relative temporal trends and interactions among tumor microenvironment parameters, not to provide precise quantitative measurements from any single experiment or clinical trial. **(A)** Concurrent strategy, **(B)** Sequential strategy, **(C)** Induction strategy. Chemo, chemotherapy; LCRT, long−course radiotherapy; RT, radiotherapy; ICI, immune checkpoint inhibitor; pCR, pathological complete response.

Based on the simulated curves from the LCRT platform (**Figure 2**), the three sequencing strategies exhibit distinctly different kinetics of the tumor immune microenvironment. **Figure 2A** (Concurrent) shows that early during RT, although rapid antigen release is induced, circulating and tumor-infiltrating T cells undergo a transient decrease due to radiation sensitivity (49, 50). Concurrent ICI administration may partially protect T cells from radiation-induced apoptosis, but T-cell proliferation and activation require time. Their numbers only start to recover after the initial decline, with the proliferation peak occurring after this recovery phase (49). Consequently, in the concurrent strategy, ICI administration actually precedes the main window of T-cell activation (light green shaded area in **Figure 2A**), so that by the time T cells have massively expanded and PD-1 is highly expressed, ICI blood concentrations may have already peaked or the course may be nearing its end; exhaustion levels at the end of follow-up remain moderate. **Figure 2B** (Sequential) shows that ICI is delayed until after RT completion, exactly aligning with the T-cell activation window (when T cells reach their peak), allowing ICI to most effectively reverse functional inhibition and promote tumor killing. However, during the late RT phase without ICI protection, sustained high-intensity T-cell activation leads to rapid accumulation of exhaustion markers, reaching the highest exhaustion levels among the three strategies by the end of follow-up. Residual T cells after RT are enriched for exhaustion-related molecules such as Lag3 and Tim3, and even subsequent ICI administration cannot fully restore their function (50). **Figure 2C** (Induction) shows that during the induction phase, T cells already rise substantially under ICI protection, with PD-L1 gradually upregulated (51). When RT starts later, antigen release is delayed, but T cells do not experience the early decline-rebound fluctuation seen in the concurrent or sequential strategies; instead, they continue to expand rapidly from a high baseline, reaching the highest T-cell peak among the three strategies and exhibiting a prolonged plateau phase after the peak. Because ICI coverage is continuous throughout (induction, RT, and consolidation phases), the onset of exhaustion is delayed, and exhaustion levels at the end of follow-up are the lowest. PD-L1 upregulation induced by chemoradiotherapy is transient (52), and RT also upregulates PD-L1 expression in radioresistant colorectal cancer cells (51), findings that further support moving ICI administration to before or during RT. The above dynamics are based on the LCRT platform. For SCRT, which features a shorter treatment duration and more rapid antigen release, the corresponding simulated curves are shown in **Supplementary Figure 1**.

To validate the aforementioned hypothesis, multiple clinical trials have designed various combination regimens and accumulated corresponding evidence. Representative studies of concurrent chemoradiotherapy combined with immunotherapy, such as the AVANA trial (53) aimed to evaluate the efficacy and safety of the PD-L1 inhibitor Avelumab in combination with nCRT for LARC. By administering the PD-L1 inhibitor avelumab concurrently during extended-course chemoradiotherapy (50.4 Gy + capecitabine), a pCR rate of 23% was achieved in 96 surgical patients. Another study R-IMMUNE (54, 55) (Phase Ib/II) employed a similar concurrent strategy. LARC patients received preoperative RT (45–50 Gy/25 fractions) concurrently with 5-FU chemotherapy, combined with atezolizumab (administered to Phase II patients at weeks 3, 6, 9, and 12 for a total of 4 doses). Among 37 evaluable patients, a pCR rate of 27% (10/37) was reported, meeting the Simon two-stage design criteria for continuation. Similarly, the NRG-GI002 trial (56, 57) evaluated a concurrently integrated strategy within the TNT framework. 185 stage II/III LARC patients were randomized after 4 months of FOLFOX chemotherapy to either the experimental arm (PA, n = 90) receiving pembrolizumab (200 mg every 3 weeks for 6 doses) during and after concurrent chemoradiotherapy or the control arm (CA, n = 95) receiving only concurrent chemoradiotherapy (capecitabine + 50.4 Gy RT). The NRG-GI002 trial showed that adding pembrolizumab to chemoradiotherapy did not significantly improve pCR (31.9% *vs*. 29.4%) but was associated with a significant 3-year overall survival (OS) benefit (97% *vs*. 95%).

The sequential/consolidation regimen is currently the primary direction of exploration. This strategy, backed by its outstanding efficacy data, represents the most promising approach at present. The VOLTAGE-A study (58–60) evaluated the efficacy and safety of nivolumab (240 mg every 2 weeks for 5 cycles) as consolidation therapy following nCRT (50.4 Gy RT combined with capecitabine 1650 mg/m²) in patients with LARC, followed by TME. The study found that nivolumab consolidation after standard-dose chemoradiotherapy achieved a pCR rate of 30% (11/37) in MSS patients. Biomarker analysis further revealed greater benefit in patients with PD-L1 positivity (tumor proportion score [TPS] ≥1%) or high CD8^+^/effector/regulatory T-cell ratios. Similarly, the PANDORA trial reported a pCR rate of 34.5% (19/55) with three cycles of durvalumab consolidation following standard-dose long-course chemoradiotherapy (50.4 Gy/25–28 fractions + capecitabine) in LARC patients, demonstrating favorable safety (61). The SPRING-01 trial (39) aimed to evaluate the efficacy and safety of combining sintilimab with CAPOX chemotherapy after SCRT (5×5 Gy) in patients with LARC undergoing TNT. Compared with CAPOX chemotherapy alone, the addition of sintilimab significantly improved pCR rates (59.2% *vs*. 32.7%). In subgroup analyses by tumor location, sintilimab plus CAPOX demonstrated a more pronounced benefit in achieving pCR in patients with tumors >5 cm from the anal margin compared to those with tumors ≤5 cm from the anal margin (52% [95% CI 30-73] *vs* 14% [-46-18]; p = 0.0016).

Direct comparisons from randomized phase II studies evaluating different timing strategies provide critical evidence for selecting integrated treatment approaches. The POLARSTAR study (12) demonstrated that in the context of extended chemoradiotherapy, the sequential group (receiving tislelizumab consolidation after completion of all chemoradiotherapy) achieved a significantly higher pCR rate compared to the chemoradiotherapy-only control group (32.7% *vs*. 14.0%, p = 0.019). The pCR rate in the concurrent group (immunotherapy initiated on day 8 after chemoradiotherapy) was 27.1%, showing no statistically significant difference compared to the control group (p = 0.082), suggesting that sequential consolidation may offer greater advantage within the LCRT framework. Furthermore, in an exploratory single-arm Phase II study (ChiCTR2100042785) (62) evaluating organ preservation for pMMR/MSS ultra-low rectal cancer, the combination of sintilimab with nCRT demonstrated encouraging efficacy and manageable safety. The study employed LCRT (50 Gy/25 fractions) with concurrent chemotherapy, followed by two cycles of sintilimab as induction therapy. This was succeeded by 6 cycles of capecitabine or CAPOX chemotherapy combined with two cycles of sintilimab as consolidation therapy. Among 23 enrolled patients, the clinical complete response (cCR) rate reached 43.5% (10/23). Among 10 patients who subsequently underwent surgery, the pCR rate was 20% (2/10), while MPR (defined as TRG 0-1) reached 60% (6/10). Combined, the overall complete response rate (cCR + pCR) was 52.2% (12/23). It should be noted that as a single-arm exploratory study, this abstract did not provide P-values for hypothesis testing to compare groups. However, the results provide preliminary evidence for the application of immunotherapy in the traditionally insensitive pMMR/MSS population and have driven the initiation of a prospective Phase III randomized controlled trial (NCT05215379).

Additionally, induction regimens are also under investigation. The PKUCH-R04 study (63, 64) is a single-arm Phase II clinical trial evaluating the efficacy and safety of the PD-1 inhibitor camrelizumab combined with TNT in high-risk pMMR LARC patients. The study employed a sequence of induction chemotherapy plus immunotherapy (3 cycles of CAPOX + camrelizumab) followed by long-course chemoradiotherapy (50.6 Gy/22 fractions + capecitabine) and consolidation chemotherapy (2 cycles of CAPOX). Among 21 surgically treated patients, a pCR rate of 33.3% (7/21) was achieved, with an overall cCR rate of 48% (12/25), providing preliminary evidence for this treatment model.

The three sequencing strategies each have their own advantages and disadvantages. The concurrent strategy is operationally simple but makes insufficient use of the T-cell activation peak and results in moderate exhaustion. The sequential strategy aligns ICIs around the T-cell activation peak, achieving a pCR rate of 30–35% (significantly higher than concurrent and control), but at the cost of severe exhaustion due to lack of protection during the peak. The induction strategy provides continuous ICI coverage, yielding the highest T-cell peak and the lowest exhaustion, with the greatest pCR potential (up to 36%), yet requires a longer treatment duration. Currently, the sequential strategy, supported by solid randomized controlled evidence (POLARSTAR) and without extending the RT-surgery interval, has become the mainstream approach for combining ICIs with LCRT. However, the induction strategy shows superior exhaustion control and preservation of T-cell function in terms of tumor microenvironment dynamics, making it particularly suitable for immunologically cold tumors (such as pMMR/MSS LARC) and a promising future direction. In clinical practice, the sequencing strategy should be individualized based on the patient’s tumor immune phenotype (baseline CD8^+^ T-cell infiltration, PD-L1 expression, MSI status), performance status, and treatment goals (rapid tumor shrinkage *vs*. Long-term control). Taken together, the current evidence indicates that both induction and sequential/consolidation strategies offer clear advantages, but with different trade-offs: sequential consolidation is more practical in the LCRT setting (without extending the treatment interval), whereas induction provides superior immune preservation and the highest pCR potential at the cost of longer treatment duration. Concurrent immunochemotherapy consolidation after SCRT has emerged as the most promising clinical pathway when pursuing high response rates and organ preservation. Future efforts should further define the optimal duration of immunotherapy, compare the mechanistic differences of immune consolidation after different RT modalities, and validate the impact of various sequencing strategies on long-term survival and patient-reported outcomes through large-scale phase III trials, ultimately achieving precise timing integration of combination immunotherapy.

### 3.2 Selection of radiotherapy fractionation schemes: synergistic effects of short-course and long-course radiotherapy

The fractionation regimen of RT serves not only as a tool for local control but also, due to its distinct immunomodulatory properties, has become a key determinant of systemic efficacy in the era of combination therapy. Traditionally, the choice between SCRT (e.g., 25 Gy/5 fractions) and LCRT (e.g., 50.4 Gy/28 fractions) has primarily been based on balancing local control with toxicity. Within the traditional TNT model, landmark studies like RAPIDO (65) have demonstrated that SCRT followed by chemotherapy significantly improves pCR rates (28% *vs*. 14%) and long-term outcomes compared to long-course concurrent chemoradiotherapy. In the context of immune combination strategies, the choice between the two regimens increasingly centers on which approach more effectively activates systemic immunity and provides the optimal synergistic microenvironment for ICIs.

From a theoretical mechanism perspective, SCRT and LCRT may influence antitumor immunity through distinct pathways. The high-dose fractionation of SCRT induces intense ICD and antigen release within an extremely short timeframe, potentially generating a more potent *in situ* vaccination effect that rapidly reverses the immunosuppressive microenvironment. This makes it particularly well-suited for seamless integration with subsequent immune consolidation therapy (66, 67). Conversely, the conventional fractionation of LCRT may activate sustained interferon signaling pathways, inducing broader expression of MHC-I and PD-L1 on tumor cell surfaces. Its extended treatment window also facilitates exploration of concurrent immune integration. However, LCRT may also carry risks of prolonged immunosuppressive cell infiltration and cumulative toxicity (20, 68).

In recent years, multiple clinical studies have focused on comparing and validating the efficacy and safety of these two RT modalities combined with immunotherapy. Immuno-combination regimens based on SCRT have demonstrated remarkable pCR rates. The SPRING-01 study (39) demonstrated that sequential administration of sintilimab combined with CAPOX chemotherapy following SCRT achieved a pCR rate of 59.2%, significantly superior to the 32.7% observed in the CAPOX chemotherapy alone group. The TORCH study (13) achieved a 50% pCR rate with SCRT plus toripalimab, while the PRECAM study (38) reported an exceptional pCR rate of 62.5% with SCRT plus nivolumab in MSS patients. Phase II results from the STELLAR II trial (69–71) (Phase II/III) showed that the iTNT group receiving SCRT plus sintilimab achieved a combined complete response rate (pCR + cCR) of 45.5%, significantly higher than the 25.0% in the TNT monotherapy group (p = 0.003). Furthermore, long-term follow-up from the NCT04231552 study (72) demonstrated that SCRT followed by camrelizumab and the CAPOX regimen in MSS patients not only achieved a 48.1% pCR rate but also a 3-year disease-free survival (DFS) rate of 80.2%. The UNION phase III trial (42) directly compared SCRT combined with immunochemotherapy to standard LCRT, confirming a significantly higher pCR rate in the SCRT combination group (39.8% *vs* 15.3%, p < 0.001).

In contrast, while LCRT combined with immunotherapy also demonstrated clear improvement in efficacy, the overall increase in pCR rates was generally lower than that observed with SCRT-based regimens. For example, in the PANDORA study (61), the pCR rate was 34.5% following LCRT followed by consolidation with durvalumab. In the POLARSTAR study (12), the pCR rate in the LCRT followed by tislelizumab group was 32.7%, significantly superior to the control group receiving chemoradiotherapy alone (14.0%, p = 0.019), but its absolute value remained lower than most SCRT combination regimens. The NRG-GI002 study (57) evaluated concurrent pembrolizumab during LCRT within the TNT framework, achieving a pCR rate of 31.9%. This did not demonstrate a statistically significant improvement over the control group (29.4%, p = 0.75), although it showed potential long-term OS benefit.

Based on the available evidence, SCRT demonstrates clear advantages in combination with immunotherapy during neoadjuvant treatment. Future optimization efforts should primarily focus on innovative fractionation patterns and precise targeting of radiation fields. Regarding fractionation patterns, A prospective Phase II study (NCT05176964) explored a fractionation cycle approach for large-fraction concurrent RT (73). This involved delivering a total dose of 35 Gy in 5 fractions (7 Gy per fraction), splitting and embedding the fractions within the 3-week cycles of CAPOX chemotherapy and tislelizumab. The goal was to achieve dynamic synergy between RT and systemic therapy throughout the entire treatment course. Regarding target volume design, another prospective Phase II study (mRCAT, NCT05972655) proposed lymph node-sparing SCRT (25 Gy/5 fractions) (74) strictly confined to the primary tumor bed while sparing regional lymph nodes. This approach, combined sequentially with CAPOX chemotherapy and tislelizumab, represents a shift from anatomical coverage to functional and immune preservation. These two approaches expand the boundaries of RT-immunotherapy synergy from different dimensions, offering new insights and evidence for optimizing future treatment modalities.

### 3.3 Biomarker-driven individualized therapy

Against the backdrop of increasingly mature LARC immunotherapy-based neoadjuvant treatment, identifying high-response populations capable of predicting treatment efficacy is crucial for achieving precision medicine, maximizing therapeutic benefits, and avoiding unnecessary toxicity. Biomarkers are evolving from mere prognostic indicators into core tools guiding treatment decisions. Based on the predictive value of treatment response according to distinct molecular characteristics, LARC patients can be categorized into different potential beneficiary groups, driving clinical strategies from a one-size-fits-all approach toward personalized therapy.

Defective DNA mismatch repair function or high microsatellite instability (dMMR/MSI-H) currently represents the most definitive biomarker for predicting the efficacy of immunotherapy in colorectal cancer. Such tumors generate substantial neoantigens due to their high mutational burden, creating a highly immunogenic hot tumor microenvironment. In metastatic colorectal cancer, the Phase III KEYNOTE-177 study (75, 76) demonstrated significant survival benefits of pembrolizumab over chemotherapy for dMMR/MSI-H metastatic colorectal cancer. In LARC, retrospective data indicate that neoadjuvant PD-1 inhibitor monotherapy achieves pCR rates of 75%–100% in dMMR/MSI-H patients (77–80). The VOLTAGE-A study (59), which included 5 MSI-H patients, reported a pCR rate of 60% (3/5) following nivolumab consolidation therapy. This compelling evidence indicates that dMMR/MSI-H patients represent a clearly advantaged population for immunotherapy (monotherapy or combination). Therefore, MMR/MSI status testing should be routine screening prior to treatment for all LARC patients. However, over 90% of LARC cases are pMMR/MSS, traditionally considered immunologically cold tumors due to low mutational burden and sparse T-cell infiltration, exhibiting limited response to single-agent immunotherapy (81, 82). In recent years, the TNT strategy, combining immunotherapy with chemoradiotherapy, has reshaped the tumor immune microenvironment through RT’s *in situ* vaccination effect, offering breakthrough hope for this patient population. Multiple pivotal Phase II studies have confirmed the superior efficacy of this combination strategy in pMMR/MSS populations. The PRECAM study (38) achieved a remarkable 62.5% pCR rate in 32 MSS patients using SCRT combined with the PD-L1 inhibitor nivolumab. The SPRING-01 study (39) demonstrated that sequential administration of sintilimab combined with CAPOX chemotherapy after SCRT significantly elevated the pCR rate from 32.7% in the control group to 59.2% (p = 0.015). Concurrently, the TORCH study (13) confirmed that in locally advanced pMMR/MSS rectal cancer, the TNT regimen combining toripalimab with SCRT and CAPOX achieved an overall complete response rate exceeding 55% and a pCR rate of 50%, significantly enhancing organ preservation opportunities. Phase II results from the STELLAR II study (71) also demonstrated that adding sintilimab increased the overall complete response rate from 25.0% to 45.5% (p = 0.003). These data represent a substantial breakthrough for immunotherapy in pMMR/MSS LARC, which constitutes the majority of patients.

Beyond MSI status, research is actively seeking alternative or complementary biomarkers that can more precisely distinguish treatment efficacy. Characteristics of the tumor immune microenvironment demonstrate significant predictive value. For instance, a *post-hoc* analysis of the VOLTAGE-A study (59) revealed that PD-L1 positivity (TPS ≥1%) and a high CD8^+^/eTreg ratio (≥2.5) were significantly associated with higher pCR rates in MSS patients. Regarding genomic features, exploratory analysis from the PKUCH-R04 study (63) suggested that LRP1B gene mutations may be associated with superior tumor shrinkage and clinical response (p = 0.04), and this mutation was linked to higher tumor mutational burden and neoantigen burden. Conversely, long-term follow-up data from the NCT04231552 study (72) suggested that FGFR1–3 gene deletions may represent a potential negative prognostic factor, with no patients in this subgroup achieving pCR (0% *vs* 55.6%, p = 0.086). Furthermore, explorations of consensus molecular subtype in the VOLTAGE-A (60) study and assessments of combined positive score (CPS) in the PANDORA (61) study provide additional clues for personalized prediction. However, their clinical utility requires validation in prospective, large-scale studies.

It is noteworthy that the efficacy evaluation system itself is evolving. For example, the NCT06794099 study (83) indicated that traditional cCR assessment criteria lack sensitivity following immunotherapy combination regimens. Future approaches will require integrating liquid biopsy technologies such as analysis of circulating tumor DNA (ctDNA) to establish more precise efficacy evaluation systems. Concurrently, significant progress has been made in the personalized application of chemotherapy drugs. In neoadjuvant therapy for LARC, UGT1A1 genotype-guided dose escalation of irinotecan (combined with capecitabine and RT) significantly doubled pCR rates (from 15% to 30%). This study confirmed the value of genotype-guided personalized chemotherapy in enhancing neoadjuvant treatment efficacy, although this regimen also markedly increased the incidence of Grade 3–4 hematologic toxicity and diarrhea (84).

Based on current evidence, establishing a comprehensive biomarker-driven treatment pathway is crucial. First, MMR/MSI testing serves as the cornerstone for identifying the dMMR/MSI-H population, which clearly benefits significantly from immunotherapy (especially monotherapy or simplified combination regimens). For the vast majority of pMMR/MSS patients, an intensive treatment pathway combining immunotherapy with chemoradiotherapy should be pursued. Within this pathway, centers with the capability are encouraged to perform multidimensional biomarker testing to optimize patient selection and prognosis assessment. This includes PD-L1 CPS scoring, evaluation of the tumor immune microenvironment (e.g., CD8^+^ T-cell density, CD8^+^/eTreg ratio), and testing for potential predictive genes such as LRP1B and FGFR. Furthermore, dynamic monitoring during treatment, particularly clearance of ctDNA, has been demonstrated to correlate strongly with pCR (48, 85, 86) and provides critical guidance for efficacy assessment and decision-making regarding W&W strategies.

Taken together, biomarkers are reshaping the landscape of neoadjuvant therapy for LARC. From clearly defined dMMR/MSI-H advantage groups to pMMR/MSS populations successfully breaking through barriers via combination strategies to refined stratification based on immune microenvironment and genomic characteristics, the era of personalized treatment has arrived. One ultimate goal of biomarker-guided precision screening and treatment is to induce unprecedented deep pathological responses (such as pCR), thereby offering patients the opportunity to avoid radical surgery and preserve organ function. In the future, integrating baseline static biomarkers with dynamic monitoring data during treatment to build multidimensional predictive models will ultimately achieve the precision medicine objective of selecting the right combination strategy for the right patient at the right time.

## 4 Safety and toxicity

With the increasing adoption of RT combined with ICIs in LARC, a comprehensive understanding of treatment related AEs and irAEs is essential for safe clinical practice. In the key trials summarized in **Table 1**, the overall safety profile of neoadjuvant radioimmunotherapy appears manageable. The most common grade 3 or higher AEs are hematologic toxicities, such as neutropenia and lymphopenia, as well as gastrointestinal reactions including diarrhea and nausea, which are predominantly attributable to the chemotherapy backbone. Importantly, the incidence of grade 3 or higher irAEs is generally low, typically below 15%, and includes rash, hepatitis, pneumonitis, and colitis (53, 61, 64, 72). For example, in the SPRING 01 trial (39), grade 3 or higher AEs occurred in 33.0% of the sintilimab combination arm, comparable to the control arm at 19.4%, without unexpected safety signals. In the UNION phase III trial (42), the SCRT plus immunochemotherapy arm showed a grade 3 or higher AE rate of 29.2%, which was higher than the 27.2% in the conventional chemoradiotherapy arm but still considered acceptable. Most irAEs respond promptly to corticosteroids and appropriate supportive care, and no treatment related deaths were reported in these trials.

However, when pelvic RT is combined with ICIs, a major clinical challenge arises: the overlap of radiation enteritis (RE) and ICI-induced colitis. Both conditions may present with diarrhea, abdominal pain, hematochezia, and tenesmus, yet their management differs substantially. Therefore, a systematic approach to differential diagnosis is critical for optimal patient care.

RE is a common complication of RT for abdominal and pelvic malignancies, resulting from injury to the small intestine and, less frequently, the colon. It can be classified as acute or chronic: acute RE typically occurs during RT or within 3 months after treatment initiation, while chronic RE may present after a latency of 6–36 months and can develop up to 30 years after irradiation (87). The pathophysiology involves epithelial cell death, microvascular damage, inflammation, and progressive fibrosis. Clinically, acute RE manifests with diarrhea, abdominal pain, nausea, and tenesmus, whereas chronic RE leads to malabsorption, chronic diarrhea, intestinal obstruction, bleeding, fistulas, and perforation (88). Diagnosis relies on a history of pelvic or abdominal RT, exclusion of tumor recurrence, and imaging studies. Cross-sectional imaging with CT enterography or magnetic resonance enterography is preferred for assessing strictures, bowel wall thickening, and complications; colonoscopy helps evaluate rectal or colonic involvement. Endoscopic features include mucosal friability, telangiectasias, ulcerations, and in chronic cases, strictures (89). Treatment is multidisciplinary. Medical management of acute RE focuses on symptomatic relief with antidiarrheals, bile acid sequestrants, antibiotics for bacterial overgrowth, and nutritional support (88). Corticosteroids and 5-aminosalicylates may be used, although evidence is limited. Hyperbaric oxygen therapy and probiotics have shown some benefit (90). For chronic RE with complications such as obstruction, fistulas, or perforation, surgery (preferably intestinal resection) is indicated; however, postoperative morbidity is high, and short-bowel syndrome remains a major concern (88).

ICI-induced colitis is a common and potentially severe irAE that affects the lower gastrointestinal tract. Its incidence depends on the ICI regimen: anti-PD-1/PD-L1 monotherapy causes any-grade diarrhea in approximately 10% of patients and colitis in 2%. Clinically, ICI-induced colitis typically presents with diarrhea, abdominal pain, hematochezia, and tenesmus, usually occurring 6–8 weeks after ICI initiation but can appear later or even after treatment discontinuation. Endoscopic findings are variable, ranging from normal mucosa to erythema, erosions, ulcerations, and patchy or diffuse inflammation (91). Histopathologically, three main patterns are recognized: acute active colitis (cryptitis, crypt abscesses), chronic active colitis (architectural distortion, basal plasmacytosis), and microscopic colitis-like (increased intraepithelial lymphocytes, thickened subepithelial collagen band), with acute active colitis being the most common (44%) (92). The diagnosis relies on a combination of clinical symptoms, endoscopic evaluation, and histologic confirmation; colonoscopy with biopsies is recommended for persistent grade ≥2 diarrhea to exclude other causes and to guide therapy. Management is graded: mild (grade 1) diarrhea may be managed conservatively with loperamide and hydration, while persistent grade 2 or higher colitis requires corticosteroids (e.g., prednisone 1–2 mg/kg/day). For corticosteroid-refractory cases, biologic agents are highly effective: infliximab and vedolizumab achieve clinical remission in approximately 87% and 88% of patients, respectively, with no significant difference between the two. Budesonide can be considered for microscopic colitis-like presentations. In patients receiving ICIs combined with chemotherapy or tyrosine kinase inhibitors, the incidence of all grade diarrhea is high (17%–56%), but severe colitis remains uncommon (grade ≥3 colitis ~0.5%), suggesting that a more tailored approach with close monitoring may avoid unnecessary immunosuppression (92).

To distinguish RE from ICI-induced colitis in clinical practice, several key features are particularly helpful. Timing provides an important clue: RE typically peaks within weeks after RT, whereas ICI-induced colitis can develop after the first few doses or even months later, and sometimes after treatment discontinuation. Anatomic distribution also differs markedly: radiation injury is confined to the radiation field, usually the rectum and sigmoid colon, while ICI-induced colitis is not limited to the irradiated area and often involves the entire colon, with a predilection for the left side. Endoscopic and histologic patterns are distinct as described above: RE shows mucosal friability, telangiectasias, and fibrosis, whereas ICI-induced colitis features cryptitis, crypt abscesses, and increased intraepithelial lymphocytes. Associated symptoms provide additional guidance: RE is often accompanied by urinary symptoms such as cystitis or perineal skin changes, reflecting the pelvic radiation field, whereas ICI-induced colitis frequently coexists with other irAEs like rash, arthritis, hepatitis, or endocrinopathies. CT findings may be nonspecific, but diffuse colonic wall thickening with pericolonic stranding or ascites is more suggestive of ICI-induced colitis. A multidisciplinary approach involving radiation oncologists, medical oncologists, gastroenterologists, and pathologists is strongly recommended for patients who develop significant gastrointestinal symptoms after combined radioimmunotherapy. Prompt and accurate differentiation allows for appropriate treatment escalation, such as corticosteroids or biologics, when immune mediated colitis is suspected, while avoiding unnecessary immunosuppression in patients with pure radiation enteritis.

Overall, although the combination of RT and ICIs in LARC is generally well tolerated, clinicians must be vigilant for the overlapping toxicity of radiation enteritis and ICI-induced colitis. A structured diagnostic algorithm integrating timing, endoscopic evaluation, histopathology, and imaging is essential to guide management and optimize patient outcomes.

## 5 Organ preservation and the watch and wait strategy

Given the favorable safety profile and high response rates achieved with immune-combination regimens, attention has now turned to whether these deep responses can be translated into organ preservation. Organ preservation represents a significant advancement in LARC treatment, with its core objective being to avoid radical surgery, particularly permanent stoma creation, while maintaining oncological efficacy, thereby preserving anal function and quality of life for patients. The W&W strategy, as the primary pathway for achieving organ preservation, has evolved from clinical exploration to gradual standardization since its initial proposal by Habr-Gama et al. (93). It now faces new opportunities and challenges in the era of immunotherapy-combined neoadjuvant treatment.

The traditional W&W strategy relies on a rigorous clinical and imaging evaluation system. Patients typically present with mid-to-low rectal cancer and require comprehensive assessment, including high-resolution MRI (particularly combined with diffusion-weighted imaging), digital rectal examination, endoscopy, and biopsy, to confirm cCR following nCRT. This approach offers the advantage of sparing some patients from surgical trauma and potential permanent stoma placement, significantly improving their quality of life. Large-scale real world studies from the International Watch&Wait Database (IWWD) (94) demonstrate that patients rigorously selected for cCR and undergoing the W&W strategy exhibit a 2-year local recurrence rate of approximately 25%. However, the vast majority of recurrences can be definitively treated with salvage surgery, achieving a 5-year disease-specific survival rate of 94%, confirming the strategy’s oncological safety. However, these traditional criteria have well-recognized shortcomings. The rate of cCR after conventional chemoradiotherapy remains modest, typically between 15% and 30%. More importantly, there is a substantial disconnect between clinical assessment and true pathological status. Tools such as MRI offer high specificity but lack sufficient sensitivity, which can lead to critical misclassifications: some patients who have achieved a pCR undergo unnecessary radical surgery, while others with only near−complete remission are erroneously placed under observation, thereby increasing the risk of local regrowth. These limitations have prompted the search for more precise, biology-driven biomarkers. In this context, liquid biopsy has emerged as a powerful complement to conventional imaging. Specifically, analysis of ctDNA can detect minimal or molecular residual disease (MRD) with high sensitivity, offering a dynamic, real-time assessment of tumor burden after neoadjuvant therapy (95). MRD refers to trace amounts of tumor cells or tumor DNA that remain after treatment but are undetectable by conventional imaging (95). Prospective studies have demonstrated that integrating ctDNA-MRD monitoring substantially improves the accuracy of response evaluation for patients being considered for W&W. For example, a 2025 study of LARC patients treated with TNT found that 67% of those with detectable post-treatment ctDNA harbored residual disease requiring radical surgery, compared with only 21% of ctDNA-negative patients (p = 0.035) (96). This dual finding illustrates that ctDNA positivity significantly improves sensitivity for detecting residual tumor, while ctDNA negativity enhances specificity for identifying true complete responders (97). Furthermore, meta-analysis data indicate that ctDNA can detect molecular relapse with a median lead time of approximately 5 months before conventional imaging (97), enabling earlier salvage intervention. Consequently, incorporating ctDNA-MRD into a multimodal assessment algorithm, alongside high-resolution MRI, endoscopic evaluation, and clinical examination, is rapidly becoming a cornerstone of modern organ preservation protocols. This approach shifts the decision-making paradigm from a purely clinical−morphologic assessment toward a more precise, biology-driven framework, ultimately improving the selection of appropriate candidates for W&W and reducing the risks associated with misclassification.

The combination of ICIs with chemoradiotherapy as a neoadjuvant treatment strategy for LARC has significantly deepened and broadened the scope of pathological response, fundamentally transforming the concept of organ preservation. Its core value is primarily reflected in the following aspects: First, this strategy achieves breakthrough improvements in pCR and cCR rates. Multiple pivotal clinical studies confirm that even in pMMR/MSS patients, immunotherapy combinations elevate pCR rates to over 50%–60% (13, 38, 39), with concurrent significant improvement in cCR rates. This therapeutic leap substantially expands the pool of patients eligible for W&W strategies. Second, opportunities for organ preservation have increased substantially. Research data directly supports immunotherapy’s contribution in this regard: for example, the POLARSTAR study (12) demonstrated that adding tislelizumab significantly increased sphincter preservation rates from 70% to 88% without increasing surgical complications. In the TORCH study, approximately 43.5% of patients achieving cCR successfully transitioned to the W&W strategies. These findings indicate that immunotherapy, by inducing deeper tumor regression, transforms organ preservation from a highly selective clinical decision into an evidence-based, systematically implementable clinical pathway. Furthermore, combination strategies may enhance the quality of treatment response. The synergistic effects between RT and ICIs not only enhance short-term response rates but may also potentially reduce long-term recurrence risk by activating and sustaining tumor-specific immune memory. This provides a more robust biological foundation for extended follow-up observation.

In summary, immunotherapy-based combination treatments have propelled the LARC organ preservation strategy into a new phase. The integration of ctDNA-MRD monitoring into a multimodal assessment framework has significantly improved the accuracy of cCR prediction and reduced misclassification risks. To translate these breakthrough outcomes into standardized clinical practice, a structured pathway must be established based on multidisciplinary team meeting decision-making, incorporating novel assessment tools and long-term follow-up. Following diagnosis, LARC patients should undergo baseline testing for MMR/MSI status to guide stratification, followed by intensive neoadjuvant therapy combining immunotherapy with chemoradiation to pursue deep response. Post-treatment, cCR should be rigorously validated using a multimodal system of clinical examination, high-resolution MRI, endoscopy, and serial ctDNA analysis. Patients confirmed with cCR should enter a structured W&W regimen supplemented by close follow-up and rescue treatment protocols, ultimately maximizing organ and function preservation opportunities while ensuring oncological safety.

## 6 Future perspectives

Despite these breakthroughs, translating this encouraging short-term pathological response advantage into definitive long-term survival benefits and widespread, safe clinical practice remains fraught with critical challenges and unanswered questions. First, the long-term translation of efficacy requires urgent validation. Large-scale Phase III randomized controlled trials with extended follow-up data are urgently needed to confirm whether the improved pCR rate can be robustly translated into ultimate improvements in DFS and OS.This is the fundamental basis for establishing the new strategy as standard treatment. Second, standardization and optimization of treatment regimens require refinement. This encompasses determining the optimal duration of immunotherapy, clarifying precise dosing and sequencing when combined with different chemoradiotherapy modalities (e.g., SCRT *vs*. LCRT), and establishing standardized management pathways for rare but severe irAEs. Furthermore, achieving true personalized precision medicine necessitates deepening our understanding of the mechanisms underlying immune combination therapies. Future basic and translational research urgently requires moving beyond comparative clinical endpoints to deeply explore how different RT modalities and immunotherapy combinations differentially remodel the tumor immune microenvironment. This includes investigating their effects on T-cell receptor repertoire diversity, myeloid cell subset function, and the formation of tertiary lymphoid structures. Simultaneously, identifying novel biomarkers predictive of treatment response, such as peripheral immune cell dynamics, specific cytokine profiles, intratumoral B-cell characteristics, or novel serum markers, and integrating them with genomic features to construct multidimensional predictive models will be key to achieving precise patient selection. Furthermore, dynamic monitoring using ctDNA combined with immune-specific markers not only enables more precise assessment of pathological response but also reveals real-time tumor immune evolution under therapeutic pressure. This provides a scientific basis for decision-making regarding W&W strategies and subsequent treatment adjustments.

Looking ahead, LARC therapy will inevitably enter a new phase characterized by greater personalization, precision, and humanization. This demands that clinicians and researchers move beyond merely stacking treatment modalities. Instead, they must design treatment sequences that maximize the body’s inherent anti-cancer potential, grounded in a deep understanding of the tumor immune ecosystem. Through rigorous clinical validation, close interdisciplinary collaboration, and unwavering commitment to our core value of patient-centered care, we aim to translate this scientific breakthrough into tangible, long-term benefits for every patient. This means achieving disease eradication while preserving dignity, function, and quality of life to the greatest extent possible, ultimately ushering gastrointestinal cancer treatment into a new era where cure and quality coexist.

## 7 Conclusion

The field of LARC treatment is undergoing a profound paradigm shift driven by immunotherapy. The neoadjuvant strategy centered on ICIs combined with chemoradiotherapy has not only successfully overcome the long-standing bottleneck in pCR rates faced by traditional regimens, but more importantly, it signifies a fundamental leap in treatment objectives, shifting decisively from a focus on survival time extension toward dual endpoints that equally emphasize curative treatment and quality of life preservation. This paper systematically argues for three critical optimizations to realize this potential: In treatment sequencing, sequential/consolidation regimens demonstrate significant advantages, while induction strategies show promise for immunologically cold tumors; in RT approaches, SCRT emerges as a more competitive synergistic platform; in patient selection, diversified biomarkers beyond MSI status, such as immune microenvironment characteristics and ctDNA dynamic monitoring, are propelling treatment toward precision personalization, particularly in guiding W&W decisions. Collectively, these advances establish a robust theoretical and practical foundation for substantially enhancing organ preservation opportunities.

## Supplementary material

Supplementary Figure 1 Timeline of tumor microenvironment dynamics during SCRT−based neoadjuvant regimens. The dynamic curves are qualitative, literature−informed representations derived from published biological observations and the known treatment timeline of SCRT. They are intended to illustrate relative temporal trends, not precise quantitative measurements. **(A)** Concurrent strategy, **(B)** Sequential strategy, **(C)** Induction strategy. SCRT, short−course radiotherapy; ICI, immune checkpoint inhibitor.
