# Supplementary material for: Synergistic neoadjuvant radioimmunotherapy in locally advanced rectal cancer: mechanisms of pathologic response and the shift toward organ preservation
Source: Front Immunol. 2026 Jul 15;17:1832333. doi: 10.3389/fimmu.2026.1832333 (PMC13416103; doi:10.3389/fimmu.2026.1832333)
Supplement: Supplementary Figure 1 — Timeline of tumor microenvironment dynamics during SCRT−based neoadjuvant regimens. The dynamic curves are qualitative, literature−informed representations derived from published biological observations and the known treatment timeline of SCRT. They are intended to illustrate relative temporal trends, not precise quantitative measurements. (A) Concurrent strategy, (B) Sequential strategy, (C) Induction strategy. SCRT, short−course radiotherapy; ICI, immune checkpoint inhibitor. [file Image1.pdf]

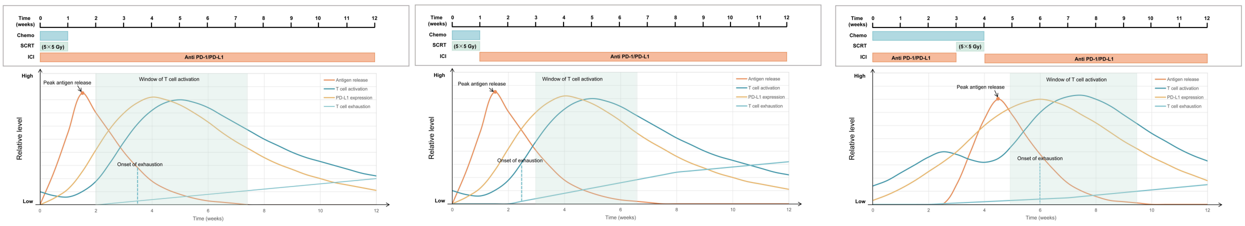

(A) (B) (C)

Supplementary Figure S1. Timeline of tumor microenvironment dynamics during SCRT-based neoadjuvant regimens. The dynamic curves are qualitative, literature-informed representations derived from published biological observations and the known treatment timeline of SCRT. They are intended to illustrate relative temporal trends, not precise quantitative measurements. (A) Concurrent strategy, (B) Sequential strategy, (C) Induction strategy. Abbreviations: SCRT, short-course radiotherapy; ICI, immune checkpoint inhibitor.
